# Public Justification and Normatively Meaningful Bias: Against imposing egalitarian accounts of algorithmic bias

**DOI:** 10.1111/bioe.70047

**Published:** 2025-11-14

**Authors:** Anantharaman Muralidharan, Julian Savulescu, Gerald Owen Schaefer

**Affiliations:** 1 Centre for Biomedical Ethics, Yong Loo Lin School of Medicine, National University of Singapore, Singapore, Singapore; 2 Chen Su Lan Centennial Professorof Medical Ethics, Centre for Biomedical Ethics, Yong Loo Lin School of Medicine, National University of Singapore, Singapore, Singapore; 3 MurdochChildren’s Research Institute, Melbourne, Victoria, Australia; 4 Uehiro Oxford Institute, University of Oxford, Oxford, UK

**Keywords:** Algorithmic Bias, Public Justification, Egalitarianism, Structural Injustice, Normatively Meaningful Bias, Responsibility

## Abstract

In a number of policy, institutional, activist and advocacy contexts, attributing bias to an algorithm does not just describe the algorithm, but impose a particular, normatively laden conception of bias on others. Given the normative content of such bias attributions, this would involve making moral demands on others to rectify the algorithm, compensate the victims of such bias and/or not unselectively deploy the algorithm. It is also the case that moral demands, especially in the abovementioned contexts are subject to a public justification requirement. As it turns out, the dominant accounts of bias in the literature presuppose some version of egalitarianism about justice and that any action that causally contributes to an unjust situation is itself wrong. Since these presuppositions are subject to reasonable disagreement, bias attributions in such situations are wrong because they violate the public justification requirement. In response, we develop a publicly justifiable conception of algorithmic bias.

## 1 Introduction

Most, if not all, accounts of the ethical deployment of algorithms, found in institutional, national and international guidelines and policies [1], posit that we need to avoid or reduce algorithmic bias. Some exemplars include the EU AI Act [2],Beijing AI principles [3], the US’s Report on the Future of Artificial Intelligence [4] and the Singapore Bioethics Advisory Committee’s consultation paper [5]. But this raises the question of what we mean by algorithmic bias. Different ways of conceptualising bias will generate different verdicts about whether an algorithm is biased and hence generate different determinations about what is to be done. Consider the following case:

Bank Loan: A bank uses an algorithm to decide whether it will issue a loan. It is found that the algorithm is less likely to issue a loan to members of ethnic minority group B than to members of the dominant group. This con-tributes to racial wealth inequality. However, after controlling for income, asset value, education and credit history, the disparity disappears. The disparity exists because members of B experience discrimination and other injustices in education, housing and in the workplace. As a result, they fare poorly on these indices and are, as such, less likely than non-members to repay a given loan.

On the one hand, some might not think it biased because the algorithm accurately assesses the likelihood of repaying a loan. On the other hand, some might think it biased because its use exacerbates certain objectionable inequalities or because the likelihood of repaying the loan is itself constituted by factors that are infected with injustice. The question of whether it is biased, hence, seems to depend on whether the algorithm is connected in the wrong way to injustice. But this still leaves us wrestling with the question of what exactly the relevant kind of connection is and what exactly justice requires. Such moralised accounts of bias may be a source of further complications, as they entail that biased algorithms should not be used or that we should make efforts to minimise or to further reduce the bias. So, to declare an algorithm biased is to put forward a moral demand, that is, a moral claim about what people ought to do. However, there might, in some circumstances, be moral constraints on what moral demands we might make. Arguably, when we have (or are trying to exert) some power over others to get them to do as we demand despite their dis-agreement, we may permissibly make a moral demand only if itis publicly justifiable. Such situations include but are not limited to the legal case where the demand has the force of a law or regulation, when drawing up guidelines for legislators and institutions, and when engaging in activism and protest. In such situations, attributions of bias are not merely descriptive but also involve an attempt to impose a certain conception of bias on others. In this paper, we will reserve the term ‘attribution of bias’ to refer to those instances that involve such an attempt to impose a certain conception of bias on others. We use the term ‘calling an algorithm biased’ to refer to the thinner merely descriptive enterprise. If attributions of bias are constrained in that way, then many of the dominant accounts of algorithmic bias violate this constraint because they presuppose controversial egalitarian accounts of distributive justice and controversial accounts of the connection between individual wrongdoing and systemic injustice. To argue for this, in Section 2, we will explore the morally charged nature of bias attributions and how they relate to certain kinds of moral demands. We will also briefly explore when and why moral demands are subject to a public justification requirement. In Section 3, we will argue that accounts of bias are structurally related to accounts of distributive justice in the following way: An algorithm is wrongfully biased only if it would result in or worsen an unjust outcome. In showing this, we will also see how the conceptions of bias that tend to be invoked in the literature rely on egalitarian conceptions of justice and such accounts can be subject to reasonable dis-agreement. In Section 4, we will argue that dominant accounts of bias presuppose a controversial account of the link between individual wrong-doing and structural injustice. Whereas these accounts presuppose that any causal contribution to creating or worsening structural injustice is wrong, other accounts of structural injustice allow that there are many instances of structural injustice in which all or most of the individual constituent actions are not wrong while the combined outcome of such actions is unjust. In Section 5, we conclude by sketching out an account of bias according to which an algorithm is biased if and only if it is publicly justifiable that using it would wrongfully cause or worsen unjust inequalities.

## 2 Normatively Meaningful Bias, MoralDemands and Public Justification Requirements

Declarative sentences sometimes do more than merely attempt to describe the world [6, 7]. They may also make moral demands. This is important as if, as we will argue, there are moral constraints on making moral demands, those same constraints apply to uttering those kinds of declarative sentences. This section will attempt to argue, first, that bias attributions, a kind of declarative sentence, involve making moral demands and, second, that such moral demands, across a range of circumstances, are subject to a public justification requirement.

### 2.1 Bias Attributions and Moral Demands

To say that an act, institution, social structure or algorithm is biased is to imply that there is at least one thing wrong with the algorithm. Indeed, to attribute bias to social structures is to demand fundamental social change. Likewise, attributions of bias are usually taken to be strong reasons to enact social change [8–11], to not use an algorithm or to only selectively deploy an algorithm [12]. Even if there are some instances where biased algorithms may still be permissibly used, we might still think that efforts must be made to improve the algorithm or perhaps compensate those whom the algorithm was biased against. One might wonder why there might not also be a merely descriptive account of algorithmic bias, some instances of which would be objectionable. On this descriptive account, an algorithm is biased_desc_ if and only if it exhibits different predictive accuracies for different classes [13]. Here, we understand predictive accuracy as the inverse of the distance between algorithm’s estimate or prediction and the ground reality.

Of course, once we adopt this account, we would no longer be able to take the mere fact that some algorithm is biased_desc_ to further imply that there is something objectionable about it and that it should either not be used or efforts should be made to reduce the bias_desc_. At least, we could not do so without the suppressed premise that all algorithms that are biased_desc_ are thereby objectionably biased. This implicitly makes reference toa normative conception of bias as necessarily objectionable, biased_norm_. An algorithm is biased_norm_ if and only if it exhibits different predictive accuracies for different classes, unselectively using it would be pro-tanto wrong and this wrong-making feature is caused by the difference in accuracy. This means that bias_desc_ is not a normatively meaningful account of bias. There will often be cases where an algorithm will be biased according to the definition of bias_desc_, but no normative conclusions can be drawn from knowing that it is biased in this descriptive sense. Arguably, in circumstances where calling an algorithm biased is ordinarily understood to have normative import, we should not call algorithms biased if they are merely biased_desc_.

To this, one might reply that perhaps we should be careful to distinguish between biased algorithms that are not objection-able and those that are. Further, we should also refrain from demanding that algorithms not be used or that bias be reduced merely on the basis of that algorithm being biased.

However, while this might seem initially like an attractive possibility, it does not reflect actual use. In a wide range of contexts including the moral evaluation of algorithms, the term has a negative connotation associated with a range of individual, distributive and structural injustices [14]. In addition, given the negative connotation of bias, we risk conceptual slippage where people mistake someone calling an algorithm biased_desc_ for calling it biased_norm_. Furthermore, if we successfully reduce or remove the normative connotation from the word ‘bias’, we risk weakening a strong social norm against objectionable bias and discrimination.

Importantly, in the kind of cases that we are interested in, namely, those involving the moral evaluation of algorithms, the relevant notion of bias is bias_norm_. When we call an algorithm biased_norm_, we imply that something must be done to reduce the degree of bias. It would indeed be very odd to claim that an algorithm is objectionably biased but there is no obligation to rectify or ameliorate this bias. A moral demand to do something, ф, is simply a directive to ф where the utterer believes that ф-ing is morally required [15]. Since calling an algorithm biased at the very least conveys that the algorithm must be improved in terms of fairness, attributing bias_norm_ involves is-suing a directive to do something believed by the utterer to be morally required. Attributing bias thus involves making moral demands. If this is right, the moral constraints that constrain moral demands would also constrain bias attributions.

### 2.2 Moral Demands and Public Justification

In situations that involve the exercise of soft or hard power, one of the moral constraints on moral demands, as has been argued by Muralidharan and Schaefer [15], is a public justification requirement. On this view, we may permissibly morally demand that someone, A, do something, ф, only if no one could reasonably disagree with the claim that A should ф. That is why it is wrong to demand that people convert to a specific religion, or keep the Sabbath, or refrain from having an abortion. Such acts are required or forbidden only under some moral and religious traditions and people can reasonably disagree with those traditions. Two people reasonably disagree about a proposition if and only if (a) the disagreement is epistemically rational, (b) would persist even when both persons reason flawlessly, (c) their evidential situation is on a par with eachother^1^ and (d) they are adequately morally motivated [15, 16].

Moral demands, in certain circumstances, are subject to a public justification requirement because it would be authoritarian, in those circumstances, to issue moral demands that people can reasonably disagree with. Such circumstances include those involving the making or enforcement of regulations and laws, institutional settings, making guidelines, activism and advocacy among others. This implies that in such public-facing contexts, when there is reasonable disagreement about what is to be done, the least demanding of the reasonable moral theories wins out. Given that many might find this implausible, it is worth saying something in defence of it: Public-facing moral demands, if they could be reasonably dis-agreed with, would be authoritarian because making such a demand on someone fails to treat them as a moral equal and instead treats them as someone who could permissibly be pushed around.

This is because demanding that someone else refrain from an action when there is reasonable disagreement about whether itis wrong attempts to impose our will on them and is hence objectionably authoritarian. When the disagreement is not reasonable, we could truly say that if they had reasoned better, or learned some crucial piece of information, they would agree with us. Then, we are merely reminding the other of the force of the moral reasons that bear on her. If so, we are merely making a permissible remonstrance to act rightly. This would be true even if the action that we demand that they refrain from indeed turned out to be wrong. What distinguishes a permissible remonstrance to act rightly from an impermissible attempt to push someone around is whether the moral truth^2^ itself or merely our will is directing the other’s actions. Since, as has been argued elsewhere [15, 17, 18], when there is reasonable disagreement, the moral demand is issued in a way that is insensitive to the moral truth, it is not the moral truth that would direct her actions were she to comply. This is what makes issuing moral demands that are subject to reasonable disagreement an attempt to impose one’s will on others and hence authoritarian. Such authoritarianism is wrong because it fails to respect the other as moral equals and treats them as being required to do something simply because it has been demanded of them.

One might worry that such a principle might seem counter-intuitive because it ends up undermining most of the legislation in any existing state. This is in turn grounded in the claim that almost every moral position could be reasonably disagreed with. Our view is less counterintuitive than it seems because it sets ahigh bar for reasonable disagreement. Since reasonableness, in our view, requires impeccable reasoning and evidential parity, certain kinds of vaccine mandates, some kinds of carbon taxes or policies to teach the theory of evolution are publicly justifiable on our account. This would be because disagreement with such policies could only occur because someone made a mistake in reasoning or lacked some crucial piece of evidence. We might illustrate this point with the question of vaccine mandates. Even libertarians, who are highly committed to personal liberty, have defended vaccine mandates on the grounds that going about unvaccinated poses an unacceptable risk to innocent others and hence violates their rights [19, 20].One might object that the risk that the unvaccinated pose toothers is less severe than other activities like driving [21].However, this is not true in some pandemics like COVID19 that were much more lethal^3^ than even drunk driving.^4^ On any reasonable conception of autonomy, it can be defeated by a sufficiently high risk to innocent others, thus justifying prohibitions on reckless behaviour like shooting into a crowd or drunk driving. Given that it would be unreasonable to weigh autonomy so highly as to permit drunk driving, it must be likewise unreasonable to weigh autonomy so highly as to permit acts that are significantly more risky than drunk driving.

Likewise, for children, refusing the standard panel of childhood vaccines (like for MMR, Polio and Pertussis) for one’s own child imposes a high risk of illness or death on them that is not comparable to other risky things like gun ownership that libertarians defend^5^. This is an imposition of risk because children cannot choose for themselves whether or not to assume the risk. Since parents do not own ^6^ their children but are merely fiduciaries for their best interests, they do not have a legitimate autonomy-based interest in deciding one way or the other about whether their child should be vaccinated. Meanwhile, the child has an autonomy-based interest in, at least, living to be old enough to exercise her autonomy. If this is right, the value of autonomy cannot justify refusal of the standard panel of childhood vaccines. Hence, at least during pandemics like COVID-19 and for very young children, vaccine mandates can be justified even on libertarian grounds and are hence publicly justifiable. Arguably similar claims can be made of some other commonsense policies. The public justification requirement, then, is less counterintuitive than people might initially think because there is a high bar for reasonable disagreement.

To be clear, not every expression of a moral claim, even when not publicly justifiable, is an attempt to impose one’s will on others. For instance, neither assertions in the philosophy classroom or many ordinary debates about morality nor a company’s CEO deciding that her company’s own algorithm is too biased for deployment would involve attempts to impose one’s will on others.

Potentially problematic moral demands are more ubiquitous than those cases that arise in the context of legal enforcement. ^7^Certainly, if research ethics committees or clinical ethics committees made bias attributions that were not publicly justifiable, that would involve at least an attempt at will imposition. Apart from these, we can also include the moral claims that we might make in engaging in activism, social media pile-ons and certain kinds of interpersonal interactions. Consider how a man who harangues a woman he considers to be skimpily dressed to ‘cover up more’ is being authoritarian even in cases where there is no threat of force and the background culture is egalitarian. Cornering a tech company CEO at a restaurant and haranguing her about the bias in her company’s algorithm would likewise also be authoritarian if people could reasonably disagree about whether it was objectionably biased. Plausibly, guidelines written by those with significant political and legal influence as well as academic papers promulgating ethical frameworks that are intended to be adopted by legislative bodies as justifications for policies are subject to the requirement. By contrast, more scholarly exploration of ideas may not [34]. Even if the dis-tinction between policy-adjacent and scholarly papers is not precise [35], there are at least some papers that are clearly policy-adjacent or that are rather explicitly written from within an activist or advocacy framework^8^ and hence subject to a public justification requirement. If at least some of the above is right, then while there is some uncertainty about the scope of the public justification requirement, it clearly applies to a great deal more situations than just the narrow legal and institutional enforcement ones. Let us focus on these situations where it does apply.

Since accusations of bias in such circumstances often implicitly suggest the imposition of a moral demand by the relevant bodies, this places a public justification requirement on many bias attributions. We ought to attribute bias to an algorithm only if (a) it exhibits different predictive accuracies for different classes and (b) no one could reasonably disagree that (bi) unselectively deploying it would be *pro-tanto* wrong and that (bii) this wrong-making feature is caused by the difference in accuracy.

What does this public justification requirement mean for our current accounts of algorithmic bias? As we argue in the next two sections, many current accounts of algorithmic bias presuppose controversial accounts of distributive justice and controversial accounts of the relationship between structural injustice and individual wrongdoing. We should attribute bias to an algorithm only when the algorithm would count as biased_norm_ according to overlapping consensuses of reasonable accounts of distributive justice and structural injustice. This is the best way to ensure public justifiability in attributing algorithmic bias.

## 3 Bias and Distributive Justice

Accounts of algorithmic bias often presuppose some account of distributive justice [38, 39]. A typical definition in the healthcare context is that an AI is biased when ‘the application of an algorithm compounds existing inequities in socioeconomic status, race, ethnic background, religion, gender, disability or sexual orientation to amplify them and adversely impact inequities in health systems’ [40]. For another, more general, example, ‘[algorithmic] bias refers to the systemic and repeatable errors in a computer system that create unfair outcomes, such as privileging one arbitrary group of users over others’ [41]. For yet another, biased algorithms are ‘models whose use in decision making may have a disproportionately adverse impact on protected classes’ [14]. Notably, each account makes some reference, either explicitly or implicitly, to questions of distributive justice.

The first makes reference to inequities, the second mentions the fairness of outcomes and the third refers to the impact on protected classes (presumably the victims of historical or structural injustice). Even so, absent some specific account of distributive justice, it is unclear why all adverse impacts on the victims of historical or structural injustice are necessarily wrong. Yet, insofar as the relevant concept of bias is indeed bias_norm_, any adverse impact on the victims of historical or structural injustice must necessarily be wrong in order for the bias_norm_ attribution to be accurate. If this is right, then a necessary condition of an algorithm being biased_norm_ is if unselectively deploying it would result in a violation of the requirements of distributive justice.

Yet, there is, at least prima facie, reasonable disagreement about what distributive justice requires. The range of views about distributive justice could be roughly grouped in the following way. There are strong egalitarian [42, 43] views according to which any inequality is always all-things-considered wrong. On moderate egalitarian [44] views, inequality is always pro tanto wrong, but not always all-things-considered wrong. For instance, gains in aggregate utility can outweigh increases in inequality. What makes such views egalitarian is that equality in utility, resources or some other distributand is valuable in and of itself. For instance, while utilitarianism is also a theory of justice that treats everyone’s well-being equally, it is totally insensitive to distributions of well-being and lends no direct priority to the worst off. It is concerned only with the total aggregate of well-being. However, concerns about the diminishing marginal utility of wealth can result in egalitarian recommendations about distribution of resources. Here, egalitarian distributions of resources are only instrumental to some other goal [45]. It is, hence, not a form of egalitarianism.

There are two reasons we might have to call egalitarianism, in either its strong or moderate forms, into question. First, as exemplified by utilitarianism, regarding each person as having equal moral worth is fully compatible with accepting any amount of disparity in well-being [45] between persons. There is, hence, no tight connection between intuitive ideas about people’s equal moral worth and equality [12] of well-being. This places doubt on there being any positive reason to think that egalitarianism is true.

Second, any egalitarian account of justice is subject to the levelling down objection. On this objection, egalitarianism has the implication that there is one thing good about an event that erased disparities in well-being by worsening the well-being of the well off without improving anyone’s well-being [45]. The real harms that people suffer in pursuing equality [12] can give us some reason to doubt that equality is worth pursuing at all.

While neither of these considerations are decisive, they do give space to the idea that there can be other reasonable accounts of justice that are not egalitarian. On such views, equality itself may only be valuable instrumentally or incidentally. For prioritarians [45, 46], what is of importance is the well-being of the worst off. Improvements to the well-being of the worst off are more morally important than quantitatively similar improvements to the well-being of the better off. In/Equality is only of instrumental concern: some ways of improving the well-being of the worst off involve reducing inequality by redistributing from the wealthy to the poor. At other times, attempts to bring about equality may harm the worst off. Yet another view is sufficientarianism [47–49], according to which, there is some threshold above which the well-being of the worst off matters just as much as the well-being of the better off. Below the threshold, the well-being of the worst off matters more. On classical liberal accounts [50–54] of distributive justice, some social safety net is justified. As such, classical liberalism may, in practice, tend to involve endorsing policy measures that prevent some people’s well-being from falling below a certain threshold. Of course, it need not do so *because* well-being that falls below the threshold matters more. Instead, the justification for policies that result in, approximately, sufficientarian distributions is that such policies are required to secure substantive freedoms or to secure against certain kinds of private domination. Finally, there are libertarian views [31, 55–57] whereby some distribution of resources is unjust only if it is *actually* the result of illicit force, coercion, theft or deception. Whether an algorithm counts as biased_norm_ will depend on the account of distributive justice. For instance, an algorithm that counts as biased under egalitarianism may not necessarily count as biased under prioritarianism or sufficientarianism.

To illustrate our point, consider the following case:

Simple Diabetic Retinopathy: An algorithm^9^ promises to improve the rate of detection of referrable (I.e severe or progressive) diabetic retinopathy. Without the aid of the algorithm, trained graders have a 90% sensitivity in detecting referrable diabetic retinopathy (RDR). That is, only 90% of those who have RDR and attended screening would be detected and referred to a specialist by a trained grader. By contrast, the algorithm has a sensitivity of 97% for members of ethnic group M and 100% for non-members. As a result, this brings about an inequality in referral rates. Non-members are referred to specialists for RDR at a disproportionately higher rate than members. Even so, in terms of absolute numbers, more members are referred to specialists with the algorithm than without.

According to strong versions of egalitarianism, the fact that the algorithm increases inequality makes it impermissible to use. Even under moderate egalitarianism, using the algorithm would be *pro tanto* wrong even if permissible, all-things-considered, since the overall gain in utility outweighed the increase in inequality. However, the mere fact that there was one thing wrong with it would generate a duty to continue developing the algorithm to reduce the inequality even if it is already deployed. By contrast, given prioritarianism, the inequality may be justified by the increase in prospects of the worst-off [59]. One might still think that if it is possible to improve the performance for members of group M even more, there is a prioritarian duty to develop the algorithm so that it can do that even after an initial deployment. Even so, the resultant situation may not be regarded as even pro tanto unjust by sufficientarian, classical liberal or libertarian accounts of justice. A sensitivity of 97% plausibly exceeds whatever threshold or minimum that people might be entitled to. Moreover, this inequality may not have arisen from any unjustified coercion or deception.

In this particular case, then, assuming that at least one of sufficientarianism, libertarianism or classical liberalism is reasonable, we should not attribute bias to the algorithm because it is not publicly justifiable that it would, if unselectively deployed, result in distributions that have at least one thing wrong and which arise from the difference in sensitivity between members and non-members of ethnic group M. This raises the question of which conceptions of justice would count as reasonable. The worry is especially sharp if we include libertarianism as a reasonable conception of justice. Broadly speaking libertarianism is so permissive that there might be few, if any cases, where there is no reasonable disagreement about bias. As such, we might worry that if libertarianism counts as reasonable, we may not even be able to attribute bias to algorithms which most of us regard as objectionably biased but which are not unjust on some libertarian accounts.

We might pose two versions of this objection. Firstly, we might worry that consistent with the public justification requirement, we may not permissibly attribute bias to any algorithm. This is because some conceptions of justice like libertarianism would be extremely permissive with regards to what is a just distribution. If such highly permissive views are reasonable, it would seem as if there would always be reasonable disagreement about whether the resulting distribution was unjust. Secondly, even if we might attribute bias to some algorithms, it would still be, given the public justification requirement, impermissible to attribute bias to other obviously objectionably biased algorithms. This, again might be because some conceptions of justice like libertarianism are too permissive. On both versions of the objection, the public justification requirement, together with some assumptions about which conceptions of distributive justice are reasonable may potentially prove too much.

With regards to the first version of the objection, we may still attribute bias to some algorithms. Consider a variant of Simple Diabetic Retinopathy except that the algorithm has a sensitivity of 89% for members of M and 100% for non-members. Suppose that since existing medical services can easily operate at sensitivity of 90%, if the algorithm falls below that for any given group, it violates the standard of care for that group. We could, on sufficientarian grounds, argue that since the sensitivity of the algorithm for members falls below what is the standard of care, their share of quality healthcare falls below an acceptable standard. Classical liberals might likewise argue that the treatment of this group falls below what would, in absolute terms, be a decent minimum standard. The libertarian can argue that given that there is a legitimate expectation that a physician will provide at least the standard of care to her patients, the failure of the physician to do this for members, if she were to unselectively deploy the algorithm, constitutes deception. Since deception is one of the paradigmatic examples of unjustified aggression, providing less than standard of care to members of M would unjustifiably aggress against them and hence violate libertarian accounts of justice. The algorithm also clearly violates egalitarian and prioritarian accounts of justice. The utilitarian can object that the unselective use of the algorithm is suboptimal because the selective use of the algorithm on non-members of M only is pareto-superior. After all, in the latter, they can retain the benefits to non-members of M while leaving members no worse off than without the algorithm and better off than the unselective use of the algorithm. There is thus an overlapping consensus that the unselective use of the algorithm is wrong. This overlapping consensus on the wrongness of the resulting distribution clears one obstacle to calling this algorithm biased. Setting aside considerations of historical and structural injustice, attributing bias to this algorithm would be consistent with the public justification requirement.

One might still worry that even if we could still permissibly attribute bias to some algorithms, this account of bias is too sparse as we may attribute bias_norm_ only to algorithms which violate a rather extreme libertarian standard of distributive justice. However, there seem to be many obviously biased algorithms which seemingly do not violate libertarian justice. For instance, suppose that an algorithm that processes loan applications would reject a black applicant but not a white applicant despite them otherwise having an identical risk profile. Alternatively, suppose that an algorithm for screening job applicants screens away black job applicants but not white ones despite both having similar qualifications and experience. Yet, in neither of these cases is illicit (relative to libertarianism) force, deception or coercion used. In these cases, the algorithm is clearly biased, yet does not seem to violate libertarian justice. If libertarianism was reasonable and permitted such acts, a publicly justifiable account of bias would not be able to account for such cases.

It is important to note that the libertarian account of justice is incomplete in a certain sense. It claims to specify only the conditions which would justify violence or coercion by the state (or other voluntary mutual protection agency). Libertarianism, as we define it, is grounded in the Non-Aggression Principle: violence, deception, or coercion is never justified unless necessary to protect against unjustified violence, coercion or deception [31,55]. It his hence silent^10^ on the wrongness of other aspects of morality like the duty to aid others or the duty to not directly discriminate. We might think that Libertarianism’s silence on this issue is problematic because it leaves open the possibility that such discrimination could be permissible. We might regard those versions of libertarianism that pronounce such discrimination permissible unreasonable, in part, because they imply that bankers and employers do nothing wrong when they discriminate as a result of using the algorithm. In addition, such views also face deeper theoretical problems. Even for those versions of libertarianism according to which such discrimination is wrong, libertarians would still have to provide an account of why some kinds of wrongs like theft, fraud, and assault justify coercive interference and other kinds of wrongs like wrongful discrimination or failure to provide easy rescue do not. If no plausible account is available then the only option is to either regard such accounts of justice as implausible and hence not reasonable or the account becomes less libertarian to the degree that it allows state intervention in order to prevent invidious discrimination.

Libertarians who think invidious discrimination permissible might argue that it is insufficient to reject such a view as unreasonable simply because it does not yield an intuitive verdict. This would be true if intuitions were all we had to go on. Yet, there are independent considerations like disrespect [60] or harm [61] about what makes invidious discrimination wrong. In order to maintain the claim that discrimination is permissible, libertarians would have to argue that neither disrespect nor harm occurs when engaging in direct discrimination or that even if they occur are not even *pro-tanto* wrong. This seems like a tall order. Meanwhile, the claim that such discrimination is not wrong is under-theorised. Libertarians tend to argue that it would be impermissible to interfere with such discrimination or that there is no *enforceable* duty [55,62,63] to refrain from discrimination. However, libertarians have provided little systematic account about why some wrongs like assault, theft and murder are enforceable, but wrongs like discrimination are not, and there is little in the way of argument of why it would not be wrong tout court^11^.

One might still worry that if, according to some reasonable view, private discrimination is an unenforceable wrong, the public justification requirement would forbid us from calling obviously biased algorithms biased. It should be noted, however, that this objection relies on the misapprehension that bias_norm_ applies only to cases which involve the coercive enforcement of norms. However, even though attributions of bias_norm_ necessarily involve at least an attempt to exercise power, this does not amount to the coercive enforcement of a norm in the sense that libertarians are concerned with. Thus, libertarians who think that private discrimination is an unenforceable wrong would say that the right response to private discrimination is informal social pressure, not coercion by the law. If this is right, then even libertarians who believe that private discrimination is, in the narrow sense, an unenforceable wrong, are committed to calling algorithms which discriminate in such ways biased. If this is right, then the public justification requirement would not, in virtue of counting such versions of libertarianism as reasonable, forbid us from calling obviously biased algorithms biased.

The egalitarian might still object that the public justification requirement prevents us from attributing bias to algorithms which are indirectly biased. After all, no matter how much an algorithm disparately impacts two different groups despite not treating them differently, the libertarian conception of justice would still regard the resultant distribution as just and hence introduce reasonable disagreement about whether the algorithm was biased_norm_.

It would indeed be a problem if a publicly justifiable normative conception of bias is incapable of accounting for any disparate impact cases. Consider, for instance, the historical use of literacy tests to deliberately disenfranchise black voters[64]. Intuitively, it seems that the people who had implemented such literacy tests acted wrongly and were blameworthy in their wrongdoing. Likewise, so are hiring practices that do not select for job-relevant competence but still have a disparate impact [65]. If, in a given situation, deliberately disadvantaging a person or group is wrong, so must inadvertently doing so. The only thing that may change is whether doing so would be blameworthy. In jurisdictions where access to photo IDs is unevenly distributed across groups, photo ID requirements would systematically disenfranchise marginalised groups [66]. Sometimes, even if well intentioned, such policies which cause a disparate impact seem intuitively objectionable.

However, libertarianism need not be so completely permissive. For instance, in many situations the party that is advantaged by the algorithm is advantaged only because some of the wealth they hold is obtained as a result of the illicit use of coercion or force. For instance, in the US, some of the wealth that at least some of the rich have is a result of the advantages they gained by their ancestors owning slaves, extra-legal intimidation by white supremacist groups and the legally enforced restrictions on black person’s rights to freely participate in the market. If at least some of such wealth is illicit then the resulting inequality that the algorithm engenders may, on libertarian accounts of historical injustice [57], also be regarded as unjust. Also, insofar as organisations use tests or other selection mechanisms that do not select for fitness for a job and also have a disparate impact [65], they are being economically inefficient and hence violate duties to their shareholders. Libertarians would likewise view such instances of discrimination as wrong. In addition, libertarians tend to regard taxation to fund welfare programmes as impermissible takings. Any inequality that results from the government’s allocation of coercively obtained resources would be unjust. If the state were to use an algorithm for such allocation decisions, libertarians would be committed to calling such algorithms biased_norm_ at least insofar as it results in any inequality. By contrast, if this resultant inequality still left people well above the relevant threshold of a decent minimum, the classical liberals or sufficientarians could not regard the algorithm as biased_norm_. If this is right, there may be many cases where libertarianism is at least as stringent as egalitarianism. In such cases, we may permissibly attribute bias to an algorithm only if it violates the sufficientarian or classical liberal account of justice.

To generalise, while we have not covered every possible account of justice, any view more extreme than the libertarian views we have covered are likely to face at least as many problems. Not only will they have more, highly implausible consequences, they will also be unable to account for why direct discrimination is permissible. They will have to claim that direct discrimination neither disrespects, nor harms the targets of such discrimination or that even if it does, there is nothing wrong with such harm or disrespect. But this seems implausible. With respect to disparate impact, they will have to account for why the instances of disparate impact that libertarianism or classical liberalism counts as wrong are not, in fact wrong. However, it is not clear what such an account would look like. This allows us to say that generally, we may permissibly attribute bias to an algorithm only if its unselective use results in a violation of distributive justice according to the most permissive of the reasonable standards of distributive justice.

## 4 Structural Injustice and the Permissibility of Contributing Actions

Libertarianism also raises the question of how the permissibility of individual actions like using a particular algorithm are linked to the existence of structural injustice. A common feature of all extant accounts of algorithmic bias is that an algorithm is biased if it causes new or contributes to existing structural inequalities. However, it is unclear why this would be the case. After all, not every act which causally contributes to ongoing or structural injustices is itself wrong. To see why, consider the following case:

Taylor Swift^12^: Taylor Swift is, at the time of writing this, the world’s highest paid female musician. She earns her income from the sale of albums and concert tickets. Swift is white, cis-gendered, heterosexual and conventionally good looking. As such, we might infer that she is the beneficiary of a number of privileges. Insofar as transphobia, homophobia, fatphobia, lookism and racism are current or ongoing structural injustices in society, Swift is a beneficiary of such injustices. Swift’s advantages make her and people like her more likely to succeed than those who may be LGBT, non-white, fat or ugly. Since she is extremely popular, her sales of tickets and albums, even if reasonably priced, serve to exacerbate unjust inequalities.

Setting aside any misgivings about the conception of justice employed here, even if it is true that ticket and album sales exacerbate injustice, it seems implausible that it is impermissible for Swift to sell albums or tickets. Perhaps Swift’s profits and wealth need to be taxed and redistributed, but the permissibility of selling tickets and albums itself seems hard to question. This gives us at least some reason to think that not every action that causally contributes to structural injustice is itself wrong.

Indeed, the major accounts of structural injustice have exactly this implication. On Young’s [67] and Haslanger’s [68] accounts, there could be structural injustice without anybody ever doing something wrong. For instance, a series of individual permissible market transactions may still create situations where a single mother, through no fault of her own is left homeless and destitute. Young calls the situation of the destitute single mother is one where she is subject to structural oppression because the system makes her vulnerable to others’ actions making her homeless. By contrast, on Estlund’s [69] account, while all structural injustices have some wrongdoing as a causal contribution, not every act that causally contributes is wrong. For instance, legislators who enact laws which mandate segregation in education and housing, as well as the voters who voted them in, acted wrongly. It is, however, unclear whether sellers of houses and real estate agents, who sold their houses at market rates distorted by such legislation acted wrongly. It is plausible that they did not. Even when laws mandating segregation are repealed, many individually permissible actions might still combine to recreate racial segregation. For instance, since other peoples’ racism would cause neighbourhoods with significant ethnic minority presence to have lower prices, people who themselves have no racial animus against minorities would prefer to sell their properties before its prices fall too much. It is implausible that people are morally required to hold on to deprecating property just to avoid de-facto segregation. Likewise, it seems permissible for people to move their children from an under-resourced public education system to a private school even if this results in de-facto educational segregation and worsens the funding situation of the public school system. The wrongdoing, on this view, lies in the original legislation whose effects are only currently unfolding or maybe even have yet to unfold and, perhaps, the voting in of those legislators. Plausibly public schools should be better funded and not in ways that are held hostage to the private choices of individual parents.

From this, it would be too quick to conclude that we will always be able to reasonably disagree about whether using the algorithm is wrong. After all, some contributions to structural injustice are wrongful. Like the legislators who enact segregation into law or racist members of society whose racist preferences and actions cause housing prices in neighbourhoods with significant ethnic minority presence to drop, some acts which cause or contribute to structural injustice are wrong. It is possible that some algorithms which causally contribute to structural injustice are more like the legislator’s than the family which moves away in order to avoid the deprecating value of their property. Whether the causal contribution is itself wrongful would, on such accounts, depend on the particular context in which the AI is being deployed and the particular causal processes connecting the structural injustice and the differential inaccuracy of the algorithm. To illustrate, consider a variant of Simple Diabetic Retinopathy.

Diabetic Retinopathy Complication^13^: The algorithm is the same as in Simple Diabetic Retinopathy. However, it is known that patients who are members of M are statistically less likely to be referred to specialists for RDR than non-members. Only 7.3% of the patients who are referred are members of M. Yet, at least 20% of diabetics are members and they are likely to suffer from Diabetic Retinopathy at higher rates. This means that any given member with RDR is significantly less likely than a non-member with RDR to be referred to a specialist for her condition. This disparity in referrals is caused by a difference in attendance of screening for RDR. This difference in screening rates is itself caused by historical and ongoing injustices. Members of M face workplace discrimination from the majority ethnic group and hence tend to be self-employed in lower-paying and more precarious gig work. As a result they can ill afford to take time off work to attend screening. Historical injustices have restricted other avenues of career advancement and has resulted in lower educational attainment and lower medical literacy. As a result, they are less informed about the importance of preventative care. Implementing the algorithm would not improve screening attendance as the underlying causes of poor screening attendance remain the same. However, of those who attend screening and have RDR, a greater number of them would be referred to specialists. However, because non-members benefit to a greater degree from the algorithm, the disparity between members and non-members would increase. The percentage of referrals who are members would drop to 7.1%.

With the above complications to the situation, we can say a few things: Firstly, there is clearly background injustice which drives the pre-existing disparity. The algorithm exacerbates the disparity. We might even assume that the resulting distribution is unjust. Even so, we can reasonably disagree about whether using the algorithm would even be *pro-tanto* wrong. After all, solving problems of workplace discrimination and low health literacy do not seem to be the kinds of tasks that should be tackled by a diagnostic tool [72,73]. One may even wonder whether it is the responsibility of the clinician or even the hospital system (as opposed to a government ministry or charitable organisation) to tackle these problems. If so, at least one reasonable view among others is that using the algorithm is not even *pro-tanto* wrong. So long as there is at least one such reasonable view, there is reasonable disagreement about whether there was anything wrong with how the algorithm contributed to structural injustice.

By contrast, using the algorithm would be, on any reasonable account, at least, *pro-tanto* wrong if the algorithm had less than 90% sensitivity for members of some group like M. If we take the unaided sensitivity of 90% to be the appropriate standard of care, then a physician’s use of the algorithm would violate standard of care. On any plausible moral account, doctors have, for different reasons, at least a *pro-tanto* duty to promote the best interests of each of their patients [74–80]. The term “standard of care” picks out the standard a physician is expected by her peers to meet if she is not to be counted as negligent in fulfilling her duty. Assuming that a physician would indeed be morally negligent if she failed to meet the standard, we can see how, on any reasonable view, doctors have at least a pro-tanto obligation to meet the standard of care or do better. Indeed, this duty is universally recognised by all professional bodies across the world [81–83]. If an algorithm delivers less than standard of care for some patients, using it for those patients would be at least *prima facie* wrong. Disagreement that there is at least one thing is wrong with the algorithm would not be reasonable, even if all things considered, the algorithm may still be permissibly used. If so, in the case where a doctor’s unrestricted use of the algorithm causes the level of care to dip below standard of care for some patients, we could permissibly attribute bias to it.

Let us put the point more generally: On some views, everyone is morally responsible for correcting or not worsening structural injustice. This is, understandably, very demanding. On other, also reasonable views, only some of the people who are causally responsible are. Since causal contribution to structural injustice would be wrong only if that involved failing to live up to one’s responsibilities, bias against a group may permissibly be attributed to an algorithm only if a) it is less accurate for that group as compared with others, b) its use results in a violation of distributive justice according to the most permissive of the reasonable standards of distributive justice and c) no one can reasonably disagree that said violation of distributive justice was caused in a way that involves the algorithm’s users shirking their responsibilities.

## 5 Conclusion

We have, in broad terms sketched out when we may permissibly attribute bias to an algorithm. In section 2, we saw that bias_desc_ was not a normatively meaningful account of bias because there were algorithms which fit the definition of bias_desc_ but which we should not call biased. Since bias attributions are subject to a public justification requirement, as we have seen in sections 3 and 4, there may be cases where an algorithm may be biased_norm_ but which we still should not attribute bias to because people may reasonably disagree that it would be wrong to use it. As such in cases where we attribute bias in order to attempt to exercise some kind of power, bias_norm_ is not a normatively meaningful account of bias either. Instead, we propose what we take to be a normatively meaningful account of bias, bias_pj_. Assuming our account of when we may permissibly call an algorithm biased is complete, we propose that an algorithm is biased_pj_ if and only if a) it is less accurate for that group as compared with others, b) its use results in a violation of distributive justice according to the most permissive of the reasonable standards of distributive justice and c) no one can reasonably disagree that said violation of distributive justice was caused in a way that involves the algorithm’s users shirking their responsibilities.

While the formal statement of bias_pj_ is rather general and does not directly specify where the boundaries of the publicly justifiable are, we have, throughout the paper, suggested that the most permissive of reasonable standards of distributive justice were a libertarian or classical liberal standard. At least, given the views we have discussed so far, the cases which the classical liberal or libertarian view would consider biased would be converged on by the other reasonable accounts of justice. These conceptions of justice would then settle what the default standard of what counts as biased_pj_. If a more permissive view could be shown to be genuinely reasonable that itself would be good reason to doubt whether the so-called “obviously biased” cases now considered not biased ought to have been considered “obviously biased” in the first place. Likewise, if some argument were to be found which would show that all of the more permissive views like classical liberalism and libertarianism were unreasonable, bias_pj_ would be more stringent and egalitarian than we are now supposing. However this goes, the reasonableness (or unreasonableness) of any given account of justice would need to be shown.

## Data Availability

The authors have nothing to report.

## Endnotes

1 Two people’s bodies of evidence are on a par with each other if and only if it is true for both persons that adding the other’s evidence to one’s own will change which doxastic attitudes it is rational for a person to have.

2 To be clear, we are not claiming that all moral truths are publicly justifiable. Rather, we are claiming that moral demands may be permissibly made only if they are robustly connected to the moral truth and reasonable disagreement (of the kind that undermines public justifiability) undermines the robustness of the connection to the moral truth. Our position is that both the moral truth and public justifiability are necessary for moral demands to be permissible.

3 The US death toll from COVID, as of 2024, is 1.2 million, most of which occurred before 2023 [22]. By contrast, the 2022 death toll from road traffic accidents is about 43 thousand [23]. Over 3 years, this still puts the total death toll from COVID at ten times that of road accidents.

4 If we compare fatality rates in the United States, the 1.2 million death toll resulted from 111 million COVID cases. This amounts to a roughly 1% lethality rate. By contrast, in 2020, in the United States, 18.5 million drunk drivers caused 11,654 fatalities, less than 30% of whom were not passengers of the drunk driver’s vehicle [24, 25]. This amounts to a fatality rate of 0.02% if we just count harm inflicted on others and 0.063% if we count everyone.

5 There are approximately 108 million gun owners in the United States. This resulted in 463 accidental deaths out of nearly 48,000 firearm deaths in 2023 [26]. This is a less than 0.05% total death rate and an approximately 0.0004% accidental death rate from firearms. By contrast, in 2024, for infants aged 6 months and below, pertussis had a 0.4% death rate [27]. The death rate from pertussis is almost 10 times that of total gun deaths and 1000 times that of accidental gun deaths.

6 Some like Engelhardt argue that we can own our children who have yet to become full persons because they are a product of our labour [28]. However, even here, Engelhardt concedes that there are limitations on this ownership. For Engelhardt, ownership is nearly complete at the pre-natal stages, especially for those embryos, zygotes and foetuses that will never become persons. By contrast, for infants and very young children, the fact that they will become persons in the future more severely limits the ownership rights that parents have over them. Parents do not have the moral right to harm or neglect their children as the future persons have rights against them doing this. When children achieve self-consciousness (i.e., personhood), Engelhardt supposes that parents may partially own their children in a way analogous to how those whom we owe contractual duties to have a partial ownership over us. Children owe their parents obedience in exchange for parental support. Yet, here too, the ownership is limited and conditional, and failing to vaccinate one’s children is the kind of parental neglect that voids such limited ownership. In any case, even on views where parents, in some fashion, have ownership over their children, this does not give them a right to neglect or harm them.

7 One might worry that the question of legal enforcement may be moot if our public justification requirement turned out to be true. The argument would be that since some versions of anarchism are reasonable, the state and hence regulations by the state are not publicly justifiable. If so, then the question of which AI systems are biased and how they should be regulated by law becomes otiose. We can concede that while regulation by the state, narrowly conceived, becomes irrelevant if the state itself is not publicly justifiable this does not make normative analysis of AI bias irrelevant. After all, on any reasonable account of anarchism, there are still decentralised and nonhierarchical ways of regulating social conduct. Reasonable anarchism, is, as such, not a theory that just permits everything, but a theory that claims certain kinds of institutional arrangements are required. Anarchist institutions could just as easily apply highly egalitarian standards in whatever decentralised, nonhierarchical way they may have to regulate social conduct [29–33]. If so, the question of which social conduct we might regulate will still arise.

8 There are too many such articles to exhaustively list all of them. Here are some exemplars that are self-consciously engaging in advocacy for some group or cause [36, 37].

9 This is adapted from the algorithm developed by Ting and colleagues [58].

10 One might try to infer that they do consider such discrimination wrong from their insistence that market forces will tend to eliminate such direct discriminatory practices.

11 While Vallentyne [56], the sole exception, argues that discrimination, is not intrinsically wrong, actual instances of discrimination are wrong because it results in harms and an inegalitarian distribution of well-being.

12 This is inspired by the Wilt Chamberlain example in Nozick’s Anarchy, State and Utopia [57]. However, instead of concluding that redistribution is wrong, we are simply claiming that even if redistribution was justified, Swift’s original actions are not wrong.

13 These details are adapted from information found in Sia et al. [70]. and Muyskens et al. [71].
